# Analysis of the Characteristics of Cross-Regional Patient Groups and Differences in Hospital Service Utilization in Beijing

**DOI:** 10.3390/ijerph19063227

**Published:** 2022-03-09

**Authors:** Yu Yang, Yong Wang

**Affiliations:** 1 State Key Laboratory of Resources and Environmental Information System, Institute of Geographical Sciences and Natural Resources Research, Chinese Academy of Sciences, Beijing 100101, China; coral-yang@foxmail.com; 2 College of Resources and Environment, University of Chinese Academy of Sciences, Beijing 100049, China

**Keywords:** cross-regional patients, spatial characteristics, interdisciplinary, hospital service utilization, distance decay

## Abstract

When medical diagnostic difficulties occur at local hospitals, seeking high-quality services across regions becomes a priority for many patients. Traditional statistical methods in health care are unable to account for spatial characteristics such as outflow place or distributions of disease type and patient ages in the context of an increasing number of cross-regional groups; thus, these methods are incapable of studying service utilization differences among hospitals. From a geographic perspective, we analyzed the spatial characteristics of cross-regional patient groups who travelled from other places to Beijing and the spatial decay patterns in the actual service utilization of different hospitals in Beijing by using geographic calculations, geographic visualizations, and distance decay functions. We found the following results: (1) It is feasible to study patients’ cross-regional mobility from a geographical perspective. Through interdisciplinary integration, we can explore laws and conclusions that cannot be examined by traditional statistical methods in health care. (2) The characteristics of cross-regional patients who travelled from other places to Beijing were as follows: (a) Most patients came from northern China, and neoplasm treatment was the main demand of cross-regional patients; (b) patients 40–65 years old were the main cross-regional treatment group, and the average age of patients from northern regions and certain eastern coastal cities was relatively high. (3) The exponential distance decay function was the best of all five distance decay functions in fitting the distribution of cross-regional patient mobility to hospitals of different levels, types, and functional areas. The results of applying this function and the centrality calculation method showed that hospital service utilization was least affected by distance and that average radial distances (AR) were greatest in tertiary hospitals (distance decay coefficient *β* = 0.0786, AR = 664.70 km), traditional Chinese medicine hospitals (*β* = 0.0752, AR = 743.52 km), and hospitals in urban extension areas (*β* = 0.0782, AR = 693.29 km). Our results can serve as a reference for research concerning the allocation of medical resources and patients’ choices regarding medical treatment.

## 1. Introduction

Cross-regional treatment of patients is a ubiquitous medical phenomenon that reflects the status of medical reform, legislative changes, and medical system development within a country or region [1]. Patient mobility and public health are gradually becoming common concerns in multiple disciplines, such as the fields of public health, geography, planning, and sociology, thus providing an opportunity for interdisciplinary studies concerning patients’ mobility with respect to medical treatment.

In China, cross-regional treatment of patients usually refers to medical behaviors in which the medical location of a person with medical insurance is inconsistent with the insured region. Among these situations, the most common is that patients leave their local provinces and move to metropolises, such as Beijing and Shanghai, which provide better medical resources. Existing studies have noted that more than 50% of beds in most well-known tertiary hospitals in China serve cross-regional patients [2]. Understanding the characteristics of this large group and the associated differences in hospital service utilization is of great significance for rationally allocating medical resources, providing public health services equally, and guiding patients to choose reasonable medical treatment.

In the field of public health, many studies have been performed to explore the characteristics of cross-regional medical groups. Scholars have focused on different disease types among patient groups, such as tumors [3], rare diseases [4], or digestive system cancers [5], and different single-characteristic groups, such as the labor force [6] or adults [7], to explore the relationship between patient mobility when seeking treatment and the service utilization of medical resources. They aimed to discover the factors influencing medical mobility when seeking treatment and analyze the statistical characteristics of patients’ cross-regional mobility. Various studies have confirmed that the quality of medical care, the distance to medical treatment, and the age and disease type of patients are important factors affecting cross-regional medical treatment behavior [4,8,9]. However, some studies have treated patient mobility as a binary variable of “yes” and “no” and employed traditional statistical methods in health care, such as questionnaire analysis and logistic regression [4,10], which facilitated individual analysis but could not capture spatial patterns of cross-regional mobility well. Some directly performed simple summation [5] or ratio calculations regarding cross-regional patients [1], which ignored the total amount of local population impact or actual movement. Given the lack of effective spatial analysis and expression, such results can neither accurately reflect the characteristics of cross-regional patient groups nor allow for deep analysis of the spatial differences in the context of hospital service utilization. Therefore, it is urgent to apply the discipline of geography and to make full use of geocomputing and geovisualization analysis to study treatment in the context of patient mobility. Although certain studies have used spatial autocorrelation analysis to capture the clustering patterns of patient mobility [11] and have applied geographic visualization methods to discover patient functional medical areas [12], these studies have been merely preliminary attempts and have lacked in-depth analysis to explore spatial differentiation characteristics and the causes of patient groups in terms of age, disease type, origin, and destination through interdisciplinary integration. Moreover, there remains a gap in the research regarding the characteristics of cross-regional patient groups and differences in hospital utilization. Obviously, due to the significant spatial characteristics of patients’ cross-regional mobility with regard to medical treatment, it is necessary to apply the ideas of geography and accurate spatial analysis methods to conduct interdisciplinary research and exploration. For example, the geographically weighted regression model has been used to analyze the influencing factors of patient mobility [11], and the spatial network has been used to discover the spatial spillover effects of the health economy on patient mobility [13].

The spatial behavior of patients using medical resources reflects their medical mobility, access to medical services, diagnosis, and treatment outcomes [14]. Measuring medical accessibility and exploring the potential possibility of medical service utilization can provide auxiliary information to decision-makers when analyzing the equality of medical treatment opportunities and the rationality of medical resource allocation. Existing research has obviously been insufficient with respect to analyzing patients’ cross-regional mobility and empirical research based on cross-regional patient mobility data [15]. With the rapid development of spatial technology methods, distance decay analysis has gradually become an ideal method of expressing the actual service utilization efficiency of medical resources, which has been reflected by certain related studies concerning the analysis and prediction of patient mobility [16]. By analyzing the influence of distance factors on patient mobility [16], it is possible to explore the spatial distribution of the characteristics of cross-regional patient groups and gradually develop a new method of exploring the spatial decay mode of actual hospital utilization in different application scenarios [17]. Several recent studies have conducted empirical studies, capturing the functional area of cancer care by computing distance decay functions for health care utilization [14], and found differences in medical distance decay behavior among patient groups of different ages, genders, races, and health insurance statuses [18]. However, the actual medical service utilization characteristics of patients in hospitals of different types, grades, and regions still need further empirical research.

This study analyzed cross-regional patients in the field of medical public health from a geography perspective. Cross-regional patients were the study object, and characteristics of cross-regional patient groups and differences in hospital service utilization were discussed by using geographic computing and visualization, as well as the distance decay function, which provided a paradigm for cross-integration research concerning patients’ cross-regional mobility. On the basis of understanding the cross-regional medical demand of patients, the spatial pattern of patient mobility was revealed, which provided support for optimized medical resource allocation, equal public health services, and reasonable medical choices.

## 2. Materials and Methods

### 2.1. Study Area and Data Source

This study analyzed data concerning cross-regional inpatient cases in 103 secondary and tertiary hospitals in Beijing in 2015. Beijing has some of the highest numbers of medical resources and the most advanced medical technology in China. Hospitals in Beijing meet the medical demand of the city’s inhabitants, as well as those of a large number of patients from other regions. According to the 2016 National Medical Service and Quality and Safety Report [19], the largest target of the cross-regional patient influx in China in 2015 was Beijing. Therefore, our study concerning patients’ cross-regional mobility in Beijing is representative.

**Figure 1:**
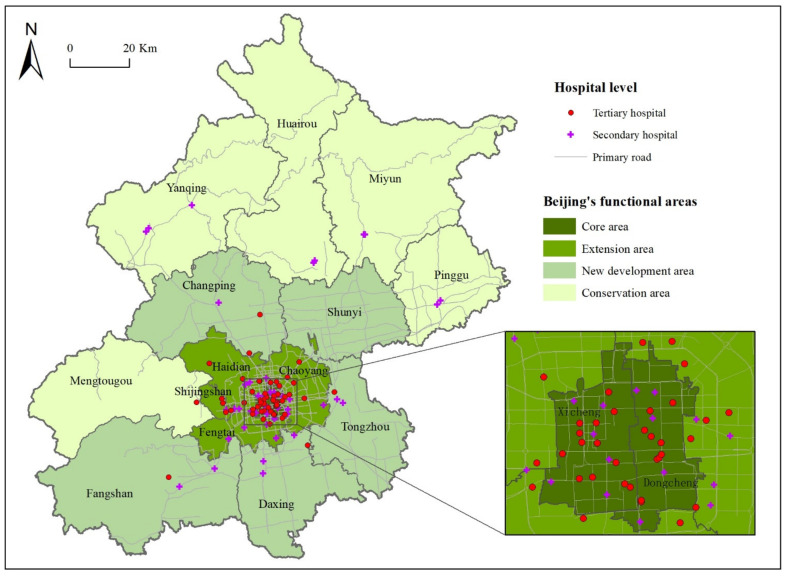
The distribution of destination hospitals in Beijing.

The 16 districts of Beijing are categorized into four functional areas (Figure 1). The core area (Xicheng District, Dongcheng District) is where the central government is located and public services are most concentrated. The extension area (Chaoyang District, Haidian District, Shijingshan District, Fengtai District) is the location of many universities and high-tech companies. The new development area (Fangshan District, Tongzhou District, Shunyi District, Changping District, Daxing District) and conservation area (Mentougou District, Huairou District, Pinggu County, Miyun County, Yanqing District) are relatively underdeveloped [20].

Patient data were collected from the health department in Beijing. The main contents of these data included each patient’s source city, age, disease type, and destination hospital. The distribution of destination hospitals is shown in Figure 1. After preprocessing, 597,952 pieces of valid data were obtained.

Administrative division data were taken from the Resource and Environment Science and Data Center, Chinese Academy of Sciences (https://www.resdc.cn (accessed on 1 August 2021)). Hospital data (including location, level, type, etc.) were obtained from the Beijing Municipal Affairs Data Resource Website (http://www.bjdata.gov.cn (accessed on 1 August 2021)).

### 2.2. Study Methods and Processes

To analyze cross-regional patient group characteristics and the different radial distances of hospital service utilization, we executed several primary processes as follows: data normalization, data spatialization, extraction of cross-regional patients, cross-regional mobility distribution fitting, and hospital utilization analysis model construction. The technical process flow is shown in Supplementary Figure S1.

#### 2.2.1. Address Positioning and Aggregation of Cross-Regional Patients

A notable feature of cross-regional mobility with respect to treatment is movement based on spatial location. The location determination of patients’ outflow sites was based on the spatialization of patients’ current addresses as described in text format. Patient original address data included vague or inaccurate information, input errors (such as typos, inconsistency of address details with provinces and cities), omissions, and inconsistent formats (standard format: county-township-village, street). To solve the above problems, we structured patient address information and mapped the address text onto a unified geographic space based on the natural language processing technology of the Baidu AI development platform (https://ai.baidu.com/tech/nlp_apply/address (accessed on 1 October 2021)) and the address processing service of Baidu Maps (https://lbsyun.baidu.com/index.php?title=webapi (accessed on 1 October 2021)). Then, we corrected any incorrect province, city, county or other fields in the address information and completed the missing geographic location information to establish an accurate spatial location for each patient. We aggregated the location of each patient in combination with the basic map unit. Addresses that could not be resolved were marked, and these locations were processed manually. Finally, based on the aggregated results of address positioning, we eliminated a few data points with unclear outflow locations and standardized and spatialized cross-regional patient data. The main process of cross-regional patient address positioning and aggregation is shown in Supplementary Figure S2.

#### 2.2.2. Calculation of Distance-Related Elements

(1) The shortest distance in terms of highway mileage from the patient’s outflow region to Beijing: The distance data from the patient’s local region to Beijing were collected from the open network platform. That is, the route planning API provided by Baidu Maps was used to extract the recommended route and calculate the shortest road mileage from the patient’s city-level region to Beijing (https://lbsyun.baidu.com/index.php?title=webapi/direction-api-v2 (accessed on 1 October 2021)).

(2) Average radial distance (AR) of hospitals in Beijing: We calculated the AR of hospitals in Beijing with Smith’s centrality calculation method [21]. That is, with Beijing as the center, every 100 km of road mileage was added as an outflow region, and the proportion of patients in each outflow region was used as a weight for calculation.

(1) $$AR=\sqrt{\left(\sum_{i=1}^{n}x_{i}^{2}d_{i}^{2}/\sum_{i=1}^{n}x_{i}^{2}\right)}$$

where *n* is the number of outflow regions, *x_i_* is the proportion of patients in the *i*th outflow region, and *d_i_* is the distance from the *i*th outflow region to Beijing.

#### 2.2.3. Hospital Patient Mobility Pattern Fitting

The term distance decay describes the geographical law that spatial interaction decreases with increasing distance. The distance decay coefficient indicates the strength of the influence of distance on the spatial interaction: the larger this coefficient is, the more significant the decay effect is with distance. There are five main kinds of functions used to fit the distance decay coefficient: the exponential model, the Pareto model, the square root model, the normal model, and the log-normal model [14]. The formulas are as follows:

(2) logI=a−βd

(3) logI=a−βlogd

(4) $$\log I=a-\beta \sqrt{d}$$

(5) $$\log I=a-\beta d^{2}$$

(6) $$\log I=a-\beta {\left(\log d\right)}^{2}$$

where *I* is the number of cross-regional patients who travel to Beijing for each city-level regional unit; *d* is the distance between the city-level regional unit and Beijing, taken as the shortest highway mileage between the city-level regional units and Beijing; *a* is a scalar factor; *β* is the distance decay coefficient. The regression coefficient of the linear model was fitted by the ordinary least squares (OLS) method, and the fitness of the model was measured by the coefficient of determination R^2^. R^2^ is the variable part of the dependent variable explained by the regression model.

R^2^ = ESS/TSS = 1 − RSS/TSS (7)

where TSS is the total sum of squares and ESS is the explained sum of squares.

## 3. Results

### 3.1. Analysis of the Characteristics of Cross-Regional Patient Groups

**Figure 2:**
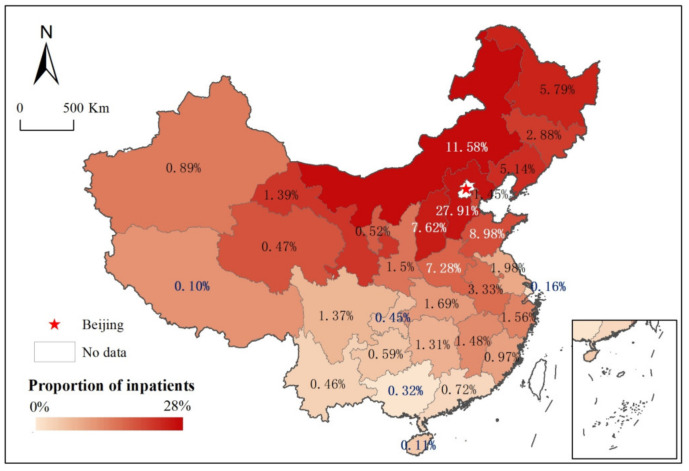
Proportion of cross-regional patients travelling from provinces across the country to Beijing.

The proportion of cross-regional patients who travelled from provinces across the country to Beijing is shown in Figure 2. The primary source of patients was the northern region around Beijing. The top 5 provinces in terms of the number of cross-regional patients travelling to Beijing were Hebei (27.91%), Inner Mongolia (11.58%), Shandong (8.98%), Shanxi (7.62%), and Henan (7.28%), accounting for 63.36% of the total patients. The bottom 5 provinces were Chongqing (0.45%), Guangxi (0.32%), Shanghai (0.16%), Hainan (0.11%), and Tibet (0.10%). The age of the cross-regional patient groups ranged from 0 to 114 years. Patients aged 40–65 were the main group, accounting for 42.88% of the total, indicating that additional attention should be given to this specific medical group consisting of middle-aged and elderly members (Supplementary Figure S3). Statistics on disease types found that the number of patients who travelled to Beijing for neoplasms and related medical services (accounting for 31.16% of the total) was much higher than the number of patients who travelled to Beijing to obtain treatment for other types of disease (Supplementary Table S1, Figure S4). Therefore, neoplasm diagnosis and treatment were the main medical demands of cross-regional patients in Beijing.

### 3.2. Differences in Cross-Regional Hospital Service Utilization

Five distance decay models were used to fit the distribution of cross-regional patients. The differences in hospital utilization at different hospital levels, of different hospital types, and in different functional areas were then analyzed. The results showed that the exponential model had the best fit (the largest R^2^) (Supplementary Table S2), which was consistent with the results of existing studies [14,22]. The fitted overall distance decay coefficient was 0.0786. Comparing the distance decay coefficients of different hospitals, we found that cross-regional patients treated in secondary hospitals (distance decay coefficient *β* = 0.0786) were more affected by distance than those treated in tertiary hospitals (*β* = 0.0786). Cross-regional patients treated in specialized hospitals (*β* = 0.0812) were more affected by distance than those treated in general hospitals (*β* = 0.0779) or TCM (traditional Chinese medicine) hospitals (*β* = 0.0752), and cross-regional patients treated in TCM hospitals were least affected. Cross-regional patients treated in hospitals in the urban extension area (*β* = 0.0782) were least affected by distance, and cross-regional patients treated in hospitals in the conservation area (*β* = 0.0816) were most affected by distance.

#### 3.2.1. Cross-Regional Hospital Service Utilization at Different Levels

**Figure 3:**
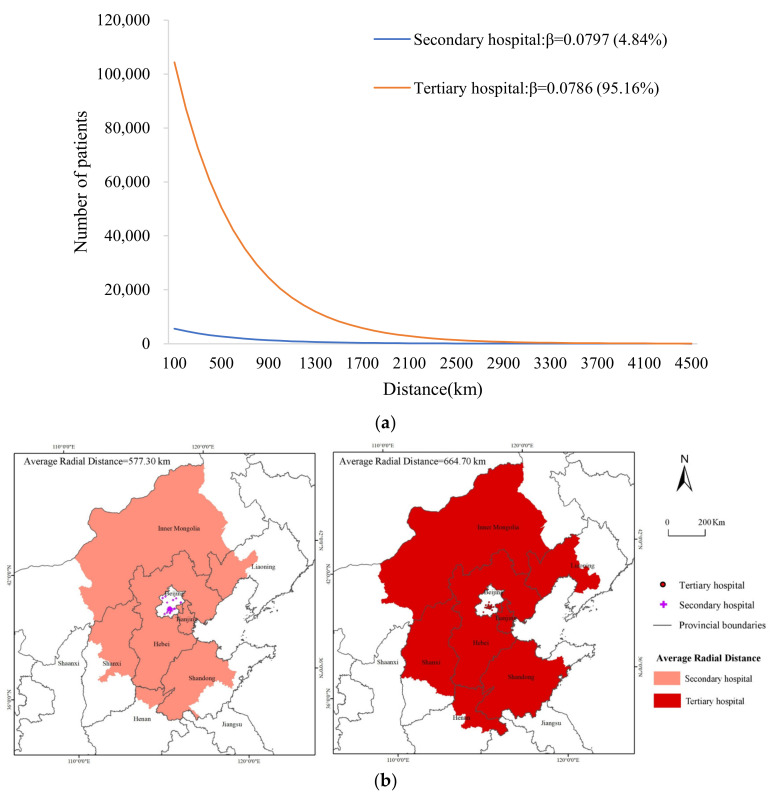
Hospital service utilization at different levels. (**a**) Fitting graph of the number of cross-regional patients according to distance in hospitals at different levels. (**b**) The AR of hospitals at different levels.

For the exponential distance decay model, the fitting results of the patient distribution patterns of hospitals with different levels are shown in Figure 3a. The declining trend of the number of cross-regional patients in tertiary hospitals (*β* = 0.0786) with distance was slower than that seen in secondary hospitals (*β* = 0.0797), indicating that the utilization of medical services in tertiary hospitals was less affected by distance. From the perspective of the service scale (Figure 3b), tertiary hospitals treated 95.16% of cross-regional patients, with an AR of 664.70 km. Secondary hospitals treated only 4.84% of cross-regional patients, with an AR of 577.30 km. In contrast, tertiary hospitals can provide services to cross-regional patients with greater intensity and scope, and they can offer higher medical quality, a more advanced medical level, and more medical resources.

#### 3.2.2. Cross-Regional Hospital Service Utilization of Different Types of Hospitals

**Figure 4:**
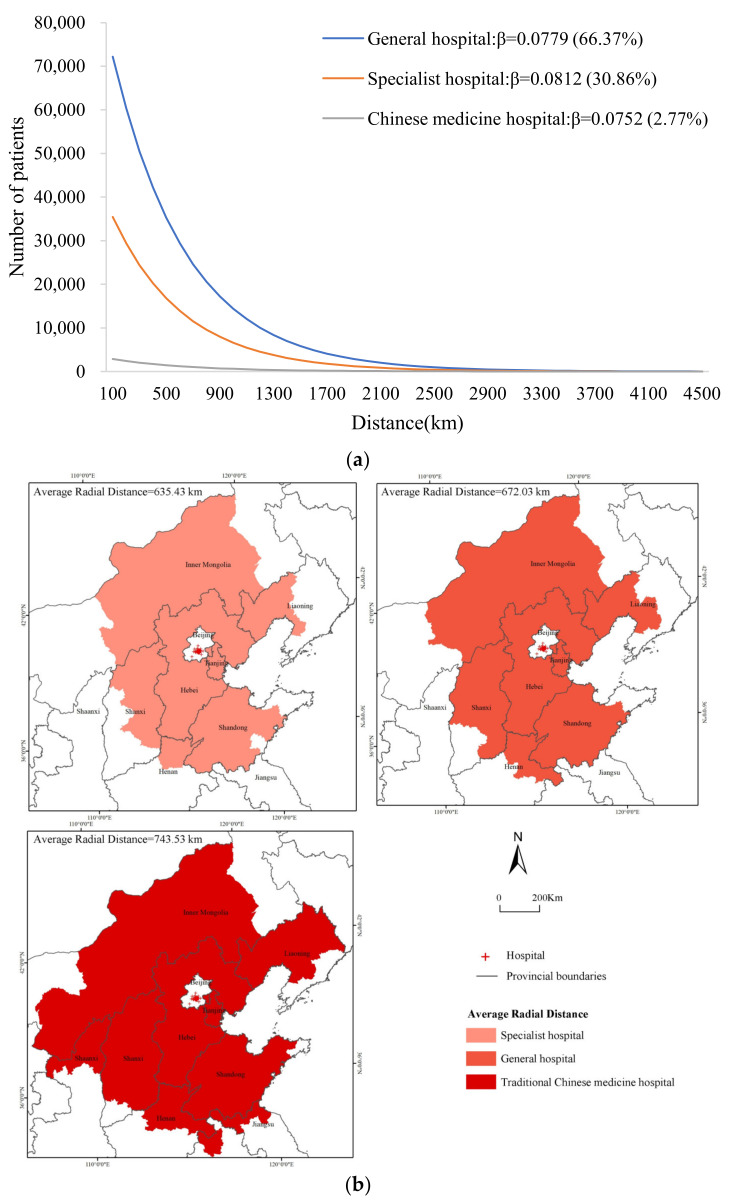
Hospital service utilization of different types. (**a**) Fitting graph of the number of cross-regional patients according to distance in hospitals of different types. (**b**) The AR of hospitals of different types.

For the exponential distance decay model, the fitting results of the patient distribution patterns in hospitals of different types are shown in Figure 4a. The declining trend of the number of cross-regional patients in TCM hospitals (*β* = 0.0752) was slower than the trend for general hospitals (*β* = 0.0779) and specialized hospitals (*β* = 0.0812), indicating that the utilization of medical services in TCM hospitals was least affected by distance. From the perspective of the service scale (Figure 4b), general hospitals treated 66.37% of cross-regional patients, with an AR of 672.03 km. Specialized hospitals accounted for 30.86% of cross-regional patients and had the shortest AR at 635.43 km. TCM hospitals only treated 2.77% of cross-regional patients from different places and had the largest AR, at 743.53 km. This finding was inconsistent with the results of previous studies concerning the flow of patients among Shanghai hospitals, which indicated that the number of patients in general hospitals declined faster with distance than that of specialist hospitals [16]. This inconsistency may be related to the study subjects and the scope of patient mobility (the difference between the Jiangsu, Zhejiang, and Shanghai patients treated in the Shanghai hospitals and the national patients treated in Beijing hospitals).

#### 3.2.3. Cross-Regional Hospital Service Utilization in Different Functional Areas

**Figure 5:**
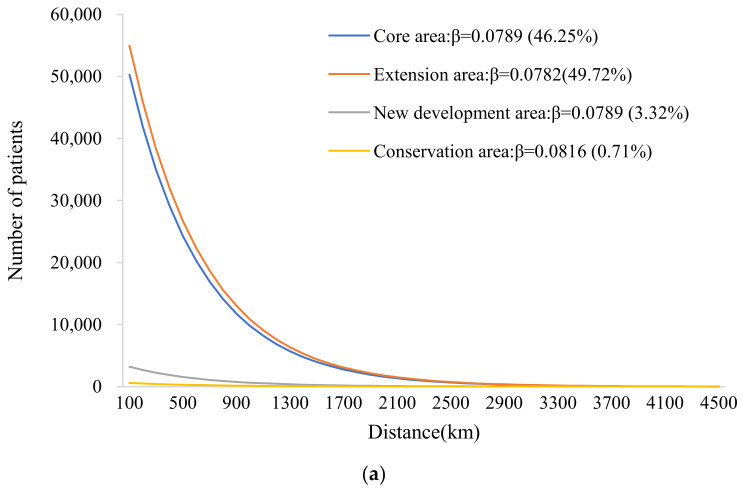

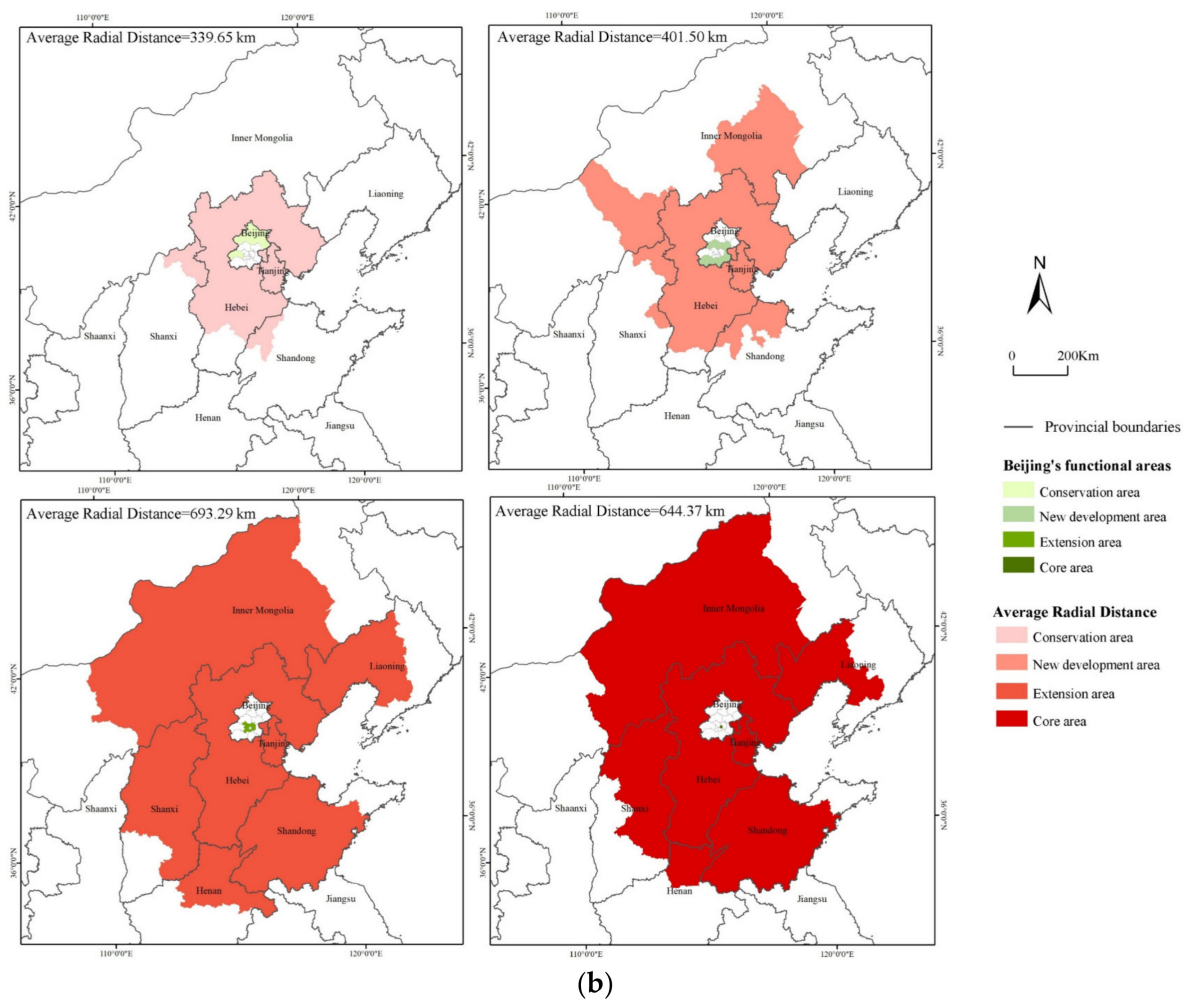
Hospital service utilization in different functional areas. (**a**) Fitting graph of the number of cross-regional patients according to distance in hospitals in different functional areas. (**b**) AR of hospitals in different functional areas.

For the exponential distance decay model, the fitting results of the patient distribution patterns of hospitals in different functional areas are shown in Figure 5a. The number of cross-regional patients in urban conservation area hospitals (*β* = 0.0816) declined faster than that in core area hospitals (*β* = 0.0789), extension area hospitals (*β* = 0.0782), and new development area hospitals (*β* = 0.0789), indicating that hospital utilization in the conservation area was most affected by distance. From the perspective of the service scale (Figure 5b), hospitals in the extension area treated 49.72% of cross-regional patients and had the largest AR, at 693.29 km. Hospitals in the conservation area treated only 0.71% of cross-regional patients and had the shortest AR, at 339.65 km. The core area and extension area of Beijing were the most concentrated areas in which cross-regional patients sought medical treatment, a fact that was closely related to the centralized distribution of high-quality medical resources in these areas. In this study, approximately 71% of Beijing hospitals were located in core and extension areas, and the quality of these hospitals was relatively high. Taking the distribution of tertiary hospitals as an example, more than 50% of such hospitals were located in Dongcheng District and Xicheng District.

## 4. Discussion

### 4.1. The Attractiveness of High-Quality Medical Resources to Patients of Different Ages

Existing studies have revealed significant differences in the medical treatment behavior of patients of different ages. For example, families with child patients are more inclined to invest resources in obtaining cross-provincial diagnosis [4], and young people are more inclined to seek out high-level medical institutions for medical treatment [23]. However, there have been few studies concerning the complex interaction between patient group characteristics and regional factors in cross-regional medical treatment.

**Figure 6:**
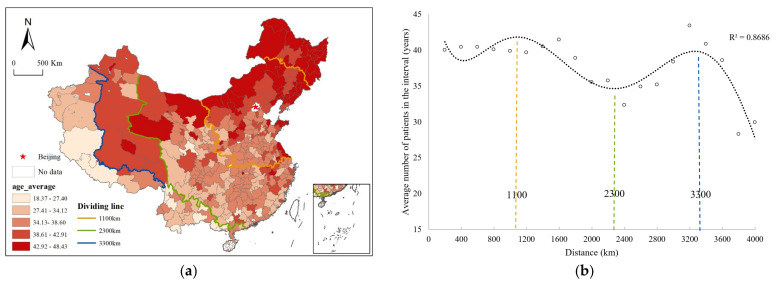
Spatial characteristics of the cross-regional patients’ age. (**a**) The average age distribution of patients travelling from each city to Beijing. (**b**) The scatter plot of distance and average age.

In this study, we calculated the average age of patients who travelled to Beijing for treatment from each city and then obtained the average age of hospitalized patients in every 200-km interval to explore the influencing factors related to the spatial differentiation characteristics of patient age. The results showed that the average age of patients from the northern region and certain eastern coastal cities was generally higher (Figure 6a). With an increase in distance from Beijing, the average patient age showed a fluctuating trend (Figure 6b). Distances of 1100 km, 2300 km, and 3300 km were identified as three significant turning points. This result may be because there are large hospitals in the Yangtze River Delta, Chengdu-Chongqing, Chang-Zhu-Tan, and Pearl River Delta regions within a range of 1100~2300 km, which have a strong ability to attract surrounding patients. A considerable proportion of elderly patients may be attracted to large medical centers closer to their local regions, so the average age of patients in this region gradually decreases with increasing distance. The 2400~3300 km range primarily includes most of Hainan, Yunnan, and Xinjiang. As the distance increased, the average age of patients gradually increased, which indicated that Beijing’s high-quality medical resources were increasingly attractive to older patients in the region. To be 3300 km away mainly included regions in Xinjiang and Tibet close to the border. The number of patients from this region was relatively small, and most of these patients suffered from major and rare diseases, indicating that Beijing’s high-quality medical resources were more attractive to young patients in the region. There is also a possibility that some elderly groups lack information and resources to access high-class medicine in Beijing. This study revealed that cross-regional patients from different age groups were affected by multiple factors, such as distance, the presence of other medical centers, and their own health conditions, thus exhibiting differentiated medical service needs. Further research is expected to obtain more data, and an in-depth analysis of the medical service utilization characteristics of different types of cross-regional patients can subsequently be conducted to provide a basis for improving regional resource allocation and ensuring a reasonable distribution of hospital services.

### 4.2. Several Potential Factors Affecting Patients’ Cross-Regional Mobility

Based on a research framework on the influencing factors of medical tourism [12], we selected four factors, namely, availability, affordability, accessibility, and economic level, and analyzed their correlation with patients’ cross-regional mobility. The dependent variable was the patient rate, that is, the number of patients who visit Beijing across regions per 10,000 people of each city-level unit. Among the independent variables, availability was constructed by using the entropy method for the number of hospitals, beds, and doctors in the city where the patient was located. Affordability was the proportion of patients’ medical insurance payments, which was used to reflect the affordability of households in the area. Accessibility was represented by the road mileage from the patient’s city to Beijing. The economic level was represented by the GDP per capita of the patient’s city.

**Figure 7:**
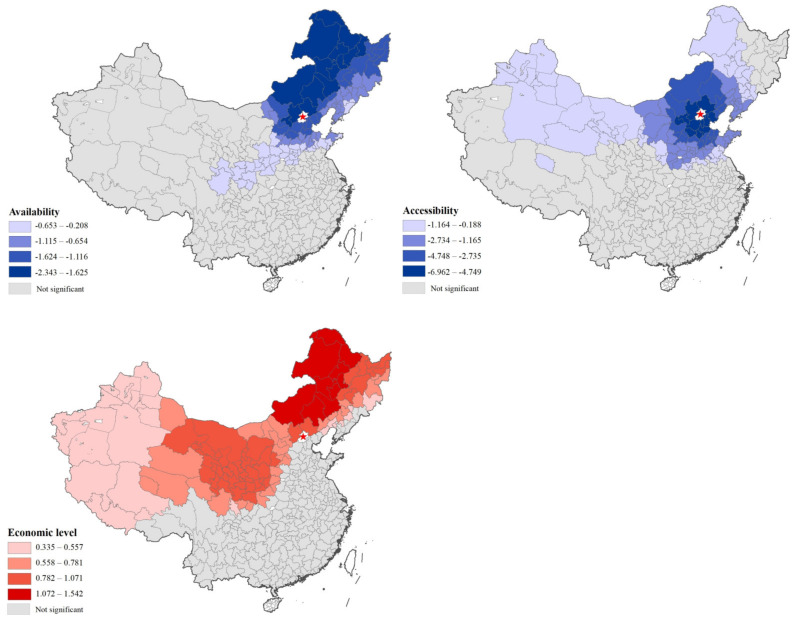
Spatial variations of local standardized coefficients to patient rate.

Table 1 shows that the GWR model reflected 81% of the change in patient rate, much higher than the 32% of the OLS model. Standardized local coefficients for three significant variables identified by the model, namely, availability, accessibility, and economic level, were mapped to reveal how these variables affected spatial variation in patient mobility (Figure 7). The impact of these variables on patient mobility varied spatially, illustrating the spatial inequality of medical level and economic development among regions.

Local medical availability was negatively correlated with cross-regional patient rates. The level of local medical care in parts of the northeast has apparently forced many local patients to travel to Beijing to receive high-quality medical care. For these special areas, the actual medical needs of cross-regional patients should be fully considered in health planning and medical center construction. Promoting the establishment of high-level hospitals will encourage more patients to receive medical treatment in their province.

Accessibility to Beijing exerted a negative impact on the cross-regional patient rate. In the cities adjacent to Beijing, accessibility exerted a great impact on patient mobility. This positive effect showed a decreasing trend from Beijing to the outside, which could be understood as the cross-regional medical effect generated by the high accessibility existing only in a small area.

The economic level was positively correlated with the cross-regional patient rate, and there were two high-value areas in northern and southwestern Beijing. This result also showed the contradiction between the economic level of these regions and the needs of patients for medical treatment. It is recommended that relevant medical institutions in these regions improve the level of medical services related to basic diseases, which improves the possibility of local diagnosis and treatment for patients with common diseases.

### 4.3. The Nationality of Beijing Medical Services and the Regional Distribution of Resources

**Figure 8:**
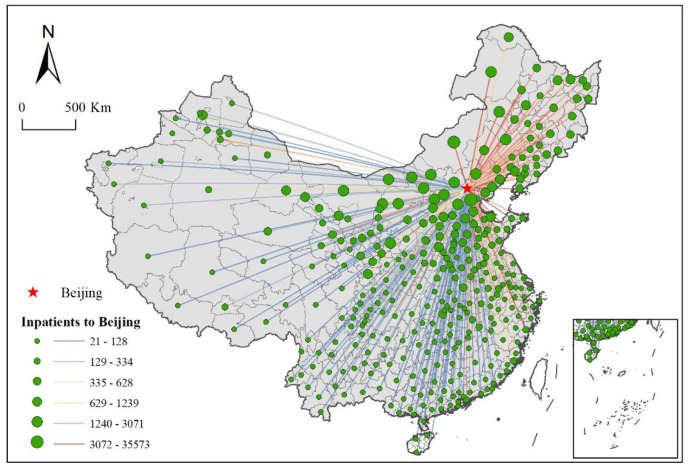
The flow of patients from city-level regions to Beijing.

The highest-quality medical resources in China are concentrated in Beijing, which has produced a siphon effect nationwide, and patients countrywide expect to travel to Beijing for treatment. As shown in Figure 8, patient movement data are visualized as a flow chart of medical visits. With a wide range of medical services, Beijing has treated patients countrywide, which reflects Beijing’s status as a national medical service center.

Nonetheless, the distribution of medical resources in Beijing is uneven, and high-quality medical resources are concentrated mainly in the central urban area. And Beijing’s high-quality medical resources with respect to neoplasms are most attractive to cross-regional patients. According to the statistics for hospitals utilized by these neoplasm patients, five hospitals—the Cancer Hospital of the Chinese Academy of Medical Sciences, Beijing Cancer Hospital, Peking Union Medical College Hospital, Peking University People’s Hospital, and Beijing Children’s Hospital affiliated with Capital Medical University—treated more than 50% of patients. All of these hospitals are located in the city center. This proximity has increased the concentration of local patients (mainly in extension areas and conservation areas) and patients from other places, which not only increases the time cost to patients but also places great pressure on traffic and social security management in the city center.

At present, in the context of China’s recent medical reform, the government promotes hierarchical diagnosis and treatment and medical alliances in which residents are encouraged to choose appropriate medical institutions according to the severity of their illness, and doctors practice medicine jointly in the context of medical alliances. These measures promote a rational and orderly medical treatment pattern [24]. When formulating medical policies, the differences in the utilization of hospital services among different hospitals need to be considered, especially for tertiary, comprehensive hospitals located in the core and extension areas, which serve more cross-regional patients with a greater average radial distance and are less impacted by distance. For example, Beijing Children’s Hospital affiliated with Capital Medical University and Peking Union Medical College Hospital in the core area can improve the radiation capacity of medical services in the new development area by building new hospitals in the Daxing district. The Cancer Hospital of the Chinese Academy of Medical Sciences can expand its own development space and contribute to the radiation of high-quality medical resources of neoplasms at the national level by building a branch in Langfang, Hebei Province. The implementation of hierarchical diagnosis and the rational diversion of patients have changed the overall spatial pattern of cross-regional medical treatment in China. According to the 2015–2020 China National Medical Service and Medical Quality and Safety Report, since 2016, Shanghai has replaced Beijing as the province with the largest inflow of cross-regional medical treatment in China. This transformation reflects the diversity and complexity of patient mobility and the rich connotations (political, economic, psychological, health-based, etc.) underlying patient mobility, which also require further in-depth research.

## 5. Conclusions

Understanding the characteristics of cross-regional patients and hospital service utilization is of great significance for rationally allocating medical resources, offering equitable public health services, and providing patients with reasonable medical choices. Based on data concerning cross-regional inpatient cases in Beijing, this study conducted an in-depth study of the characteristics of cross-regional patient groups and the spatial pattern of hospital utilization. The results demonstrated the following conclusions:

(1) Introducing the ideas and methods of geography to the study of cross-regional medical problems via interdisciplinary integration can enable the full and accurate expression of the spatial patterns of patient mobility and medical resources, which cannot be achieved by traditional statistical methods in health care. Using geographic computing, visual analysis, and the distance decay function to analyze the characteristics of cross-regional patient groups can allow researchers to comprehensively evaluate the medical demand of different patient groups and their utilization of different medical services. This study also promotes the interdisciplinary integration and development of public health, geography, planning, and sociology.

(2) This crossover study found that cross-regional patients presented the following characteristics: (a) Patients were mainly from northern China, and patients from the top 5 provinces (Hebei, Inner Mongolia, Shandong, Shanxi, and Henan) accounted for 63.36% of the total. The number of patients with neoplasms and related medical issues was much higher than that of patients with other types of diseases, accounting for 31.16% of the total. Therefore, on the one hand, it is necessary to strengthen the construction of medical alliances between Beijing and the hospitals where patients mainly flow out, promote mutual recognition and sharing of inspection results, and improve the level of regional medical services. On the other hand, neoplasm treatment was the main demand of cross-regional patients travelling to Beijing. Beijing’s high-quality medical resources of neoplasm can be transferred to surrounding cities so that it can undertake the relocation project of key hospitals, which will play a positive role in hospital development and patient treatment. (b) The age of the patients ranged from 0 to 114 years old, and patients aged 40–65 years were the main group, accounting for 42.88% of the total, indicating that the middle-aged and elderly members need further attention. Patient age showed spatial differentiation characteristics, and the average age of patients in northern regions and certain eastern coastal cities was generally higher. The differentiated needs of different age groups and regional groups should be paid attention to when formulating cross-regional medical policies.

(3) The exponential model, the Pareto model, the square root model, the normal model, and the log-normal model were used to fit the distribution of patients, and the centrality calculation method was used to calculate the radial distance of different hospitals in Beijing to analyze the differences among the three modes of hospital cross-regional service utilization. The results showed that the exponential distance decay function could optimally express the cross-regional patient mobility distribution in hospitals of different levels, types, and functional areas. Among these contexts, the cross-regional utilization levels of hospitals in the tertiary (*β* = 0.0786, AR = 664.70 km), TCM (*β* = 0.0752, AR = 743.52 km), and extension areas (*β* = 0.0782, AR = 693.29 km) were least affected by distance. The method adopted in this study is worth popularizing and can be further applied to study the hospital cross-regional service model.

Based on cross-regional inpatient data in Beijing, we summarized the characteristics of cross-regional patient sources, disease types, and ages from the perspective of geography and analyzed cross-regional medical service utilization in hospitals of different levels, types, and functional areas. The results of this study provide a paradigm for research concerning hospital service areas and medical resource accessibility. Nonetheless, due to limitations arising from the time scale of the data, this study cannot provide an in-depth explanation of the competitiveness of regional medical resources and the transformation of cross-regional medical centers. In future research, by acquiring massive time-series cross-regional patient data, we will study more complex cross-regional medical problems from the perspective of patient activity-mobility systems.

## Figures and Tables

**Table 1:** Results of OLS multiple regression and GWR models.

Factor	OLS Model		GWR Model	
	*β*	*t*	$\bar{\beta }$	$\bar{\left|t\right|}$
Availability	−0.477 ***	−7.943	−0.331 **	1.750
Affordability	0.024	0.528	0.057	0.647
Accessibility	−0.477 ***	−10.383	−0.743 **	2.451
Economic level	0.418 ***	7.524	0.333 **	2.417
R^2^	0.324		0.814	
Adj. R^2^	0.316		0.771	
AICc	846.274		554.770	
Residual squares	230.401		63.311	

Note: *n* = 341, ** *p* < 0.01, *** *p* < 0.001.

## Supplementary Materials

The following supporting information can be downloaded at: https://www.mdpi.com/article/10.3390/ijerph19063227/s1, Figure S1: Flow chart of technical methods; Figure S2: The process of cross-regional patient address positioning and aggregation; Figure S3: Age composition of cross-regional patient groups; Figure S4: Statistical histogram of the number of patients with various types of diagnosis who travelled to Beijing for medical treatment across regions; Table S1: Disease classification statistics for cross-regional patients; Table S2: Fitting results of cross-regional patient distribution models in different hospitals.
