# Supplementary material for: Analysis of the Characteristics of Cross-Regional Patient Groups and Differences in Hospital Service Utilization in Beijing
Source: Int J Environ Res Public Health. 2022 Mar 9;19(6):3227. doi: 10.3390/ijerph19063227 (PMC8950230; doi:10.3390/ijerph19063227)
Supplement: Supplementary file 1 [file ijerph-19-03227-s001.zip › ijerph-1584371-supplementary.pdf]

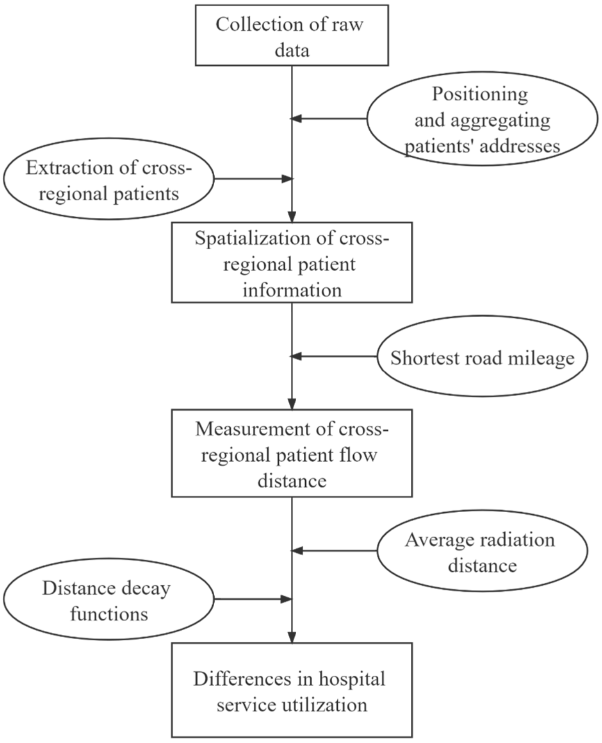

Figure S1. Flow chart of technical methods.

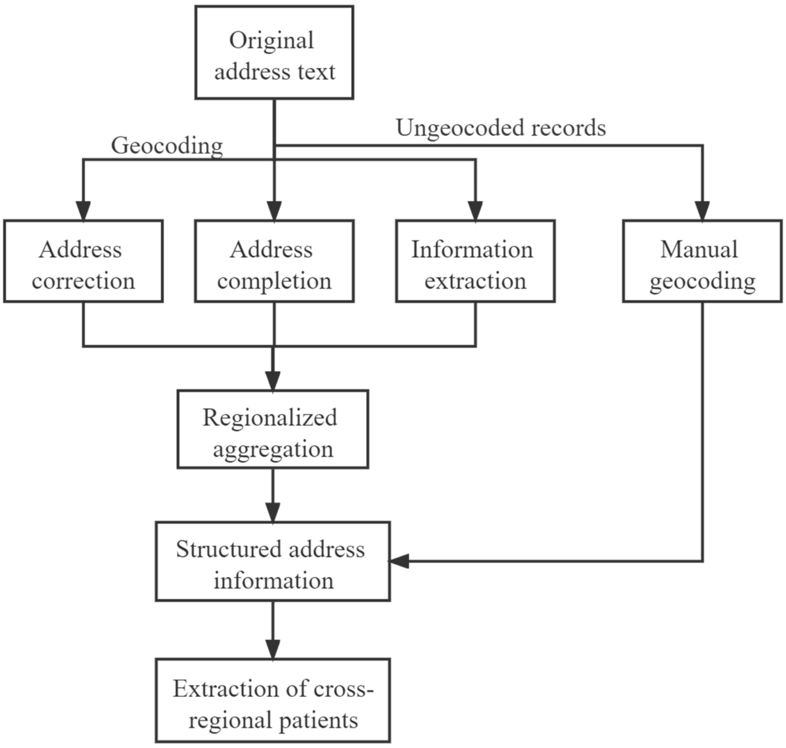

Figure S2. The process of cross-regional patient address positioning and aggregation.

The age of the cross-regional patient groups ranged from 0 to 114 years. Patients aged 40-65 were the main group, accounting for 42.88% of the total, indicating that additional attention should be given to this specific medical group consisting of young and middle-aged members of the labour force. Analysis at 5-year intervals (Figure S3) showed that most cross-regional patients were 0-5 years old, accounting for 9.29% of the total. This group was followed by patients aged 50 to 55, accounting for 9.10% of the total. For patients over 65 years old, the proportion of patients showed a significant downwards trend with increasing age, which was related to the relatively poor initiative and travel ability of elderly individuals when seeking medical care across regions.

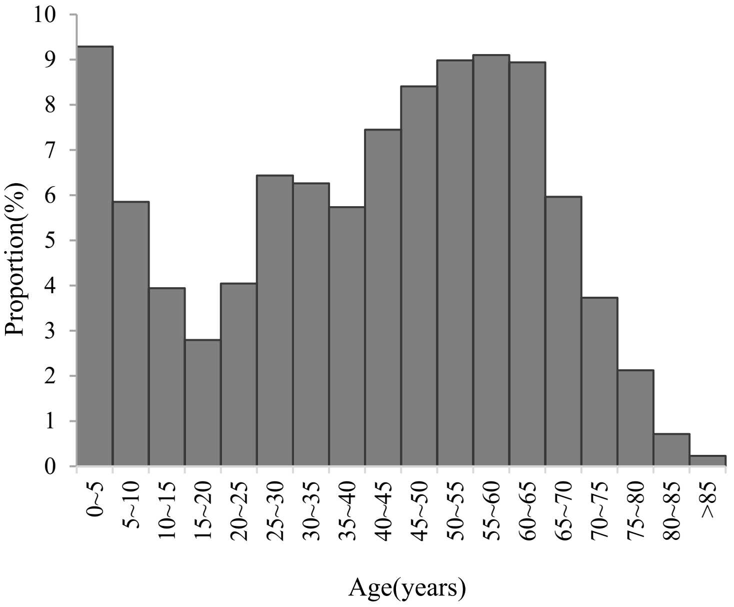

Figure S3. Age composition of cross-regional patient groups.

Diagnostic data for cross-regional patients were classified according to the ICD-10 criteria. The top 5 diagnostic types among cross-regional patients travelling to Beijing were factors affecting health status and contact with health care institutions (24.35%), neoplasms (14.76%), diseases of the circulatory system (9.71%), diseases of the musculoskeletal system and connective tissue (5.59%), and diseases of the genitourinary system (5.43%), as shown in Table S1.

Table S1. Disease classification statistics for cross-regional patients.

Code	Types	Proportion	Rank	Code	Types	Proportion	Rank
Z00-Z99	Factors influencing health status and contact with health services	24.35%	1	S00-T98	Injury, poisoning and certain other consequences of external causes	3.34%	12
C00-D48	Neoplasms	14.76%	2	E00-E90	Endocrine, nutritional and metabolic diseases	2.02%	13
I00-I99	Diseases of the circulatory system	9.71%	3	A00-B99	Certain infectious and parasitic diseases	2.00%	14
M00-M99	Diseases of the musculoskeletal system and connective tissue	5.59%	4	R00-R99	Symptoms, signs and abnormal clinical and laboratory findings, not elsewhere classified	1.64%	15
N00-N99	Diseases of the genitourinary system	5.43%	5	D50-D89	Diseases of the blood and blood-forming organs and certain disorders involving the immune mechanism	1.48%	16

H00-H59	Diseases of the eye and adnexa	5.26%	6	L00-L99	Diseases of the skin and subcutaneous tissue	1.15%	17
K00-K93	Diseases of the digestive system	5.17%	7	F00-F99	Mental and behavioural disorders	0.72%	18
G00-G99	Diseases of the nervous system	5.15%	8	H60-H95	Diseases of the ear and mastoid process	0.51%	19
Q00-Q99	Congenital malformations, deformations and chromosomal abnormalities	4.01%	9	P00-P96	Certain conditions originating in the perinatal period	0.48%	20
O00-O99	Pregnancy, childbirth and the puerperium	3.66%	10	V01-Y98	External causes of morbidity and mortality	0.00%	21
J00-J99	Diseases of the respiratory system	3.56%	11				

Although the factors affecting health status and contact with health institutions (Z00-Z99) were as high as 24.35%, this diagnostic type could not simply be analysed as a whole due to the large number of diseases and the complex health statuses involved. After further sorting and analysis, it was found that among such diagnostic codes, most medical services were related to neoplasms (including Z08, Z12, Z51.0, Z51.1, Z85), accounted for 67.33% of the total; the second most common medical services were related to reproductive medical services (including Z47), accounting for 6.78% of the total; other orthopaedic follow-up care (including Z30-Z39) accounted for 3.28% of the total. These three types of medical services were combined with their corresponding main disease categories, and all other services were regarded as one combined category, forming the histogram shown in Figure S4. The results showed that the number of patients who travelled to Beijing for neoplasms and related medical services (accounting for 31.16% of the total) was much higher than the number of patients who travelled to Beijing to obtain treatment for other types of disease. Therefore, neoplasm diagnosis and treatment were the main medical demands of cross-regional patients in Beijing.

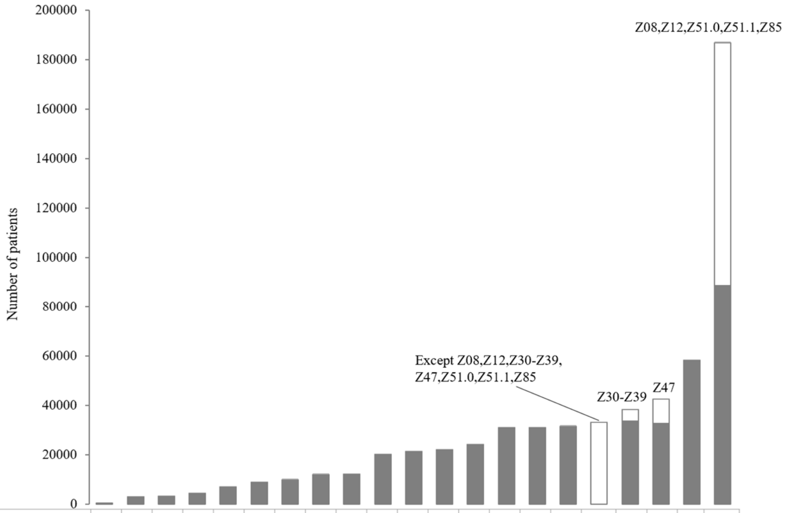

ICD-10	V01-Y98	P00-P96	H60-H95	F00-F99	L00-L99	D50-D89	R00-R99	A00-B99	E00-E90	S00-T98	J00-J99	O00-O99	Q00-Q99	G00-G99	K00-K93	H00-H59	Z00-Z99	N00-N99	M00-M99	I00-I99	C00-D48
Proportion (%)	0.00	0.48	0.51	0.72	1.15	1.48	1.64	2.00	2.02	3.34	3.56	3.66	4.01	5.15	5.17	5.26	5.51	6.39	7.08	9.71	31.16

Figure S4. Statistical histogram of the number of patients with various types of diagnosis who travelled to Beijing for medical treatment across regions.

Table S2. Fitting results of cross-regional patient distribution models in different hospitals.

		Model	α	β	R^2
Global		Exponential	5.1204	0.0786	0.8973
		Pareto	5.9411	1.9997	0.6707
		Square root exponential	6.0532	0.589	0.8308
		Normal	4.5565	0.0019	0.8799
		Log-normal	5.3463	1.1541	0.8276
Level	Secondary	Exponential	3.8238	0.0797	0.8909
		Pareto	4.6842	2.0516	0.6819
		Square root exponential	4.7718	0.5978	0.8264
		Normal	3.2604	0.002	0.8886
		Log-normal	4.0469	1.1663	0.8162
	Tertiary	Exponential	5.0971	0.0786	0.8954
		Pareto	5.9138	1.9966	0.667
		Square root exponential	6.0288	0.5889	0.8283
		Normal	4.5331	0.0019	0.8781
		Log-normal	5.3227	1.154	0.8255
Type	General	Exponential	4.9365	0.0779	0.8948
		Pareto	5.7457	1.978	0.6666
		Square root exponential	5.8591	0.5832	0.8275
		Normal	4.3784	0.0019	0.8785
		Log-normal	5.1593	1.1428	0.8243
	Specialized	Exponential	4.6307	0.0812	0.8963
		Pareto	5.4862	2.0718	0.6745
		Square root exponential	5.5968	0.6089	0.8318
		Normal	4.0478	0.002	0.8779
		Log-normal	4.8658	1.1929	0.8284
	TCM	Exponential	3.5328	0.0752	0.8637
		Pareto	4.2532	1.8445	0.6253
		Square root exponential	4.3924	0.554	0.7893
		Normal	3.0079	0.0019	0.8563
		Log-normal	3.7262	1.0839	0.7864
Functional area	Core	Exponential	4.7805	0.0789	0.8967
		Pareto	5.6138	2.0156	0.6756
		Square root exponential	5.7216	0.5925	0.8333
		Normal	4.2115	0.0019	0.874
		Log-normal	5.0112	1.1612	0.8306
	Extension	Exponential	4.8183	0.0782	0.8897
		Pareto	5.6063	1.9662	0.6486
		Square root exponential	5.7354	0.5837	0.816
		Normal	4.2619	0.0019	0.8815
		Log-normal	5.0354	1.1439	0.8132
	New development	Exponential	3.585	0.0789	0.8666
		Pareto	4.5255	2.107	0.7131
		Square root exponential	4.5619	0.6011	0.8287
		Normal	3.0058	0.0019	0.8273
		Log-normal	3.8337	1.1733	0.8192
	Conservation	Exponential	2.867	0.0816	0.8806
		Pareto	3.6494	1.946	0.6989
		Square root exponential	3.7832	0.5906	0.834
		Normal	2.3072	0.0021	0.8334
		Log-normal	3.0697	1.1513	0.8323
